# Draft genome sequences of *Lactobacillus paragasseri s*trains isolated from the female urinary tract

**DOI:** 10.1128/mra.01123-23

**Published:** 2023-12-22

**Authors:** Briana Jackson, Natalie Stegman, Cerena Sedano, Maria Steiling, Catherine Putonti

**Affiliations:** 1 Department of Biology, Loyola University Chicago, Chicago, Illinois, USA; 2 Bioinformatics Program, Loyola University Chicago, Chicago, Illinois, USA; Wellesley College Department of Biological Sciences, Wellesley, Massachusetts, USA

**Keywords:** *Lactobacillus paragasseri*, urinary tract

## Abstract

*Lactobacillus* species are often associated with a healthy environment in the female urogenital tract. Here, we present the draft genome assemblies for three *L. paragasseri* strains isolated from voided urine samples from females with type II diabetes; two of the strains were collected from the same individual 3 months apart.

## ANNOUNCEMENT

*Lactobacillus paragasseri* was first described in 2018 (1), and subsequently, many strains previously classified as *L. gasseri* were reclassified as *L. paragasseri* (2). Prior research has found urogenital *L. gasseri* strains with antimicrobial properties (3), although testing of *L. paragasseri* strains (or the resolution of species designations for previously tested strains) has yet to be published. To further investigate the antimicrobial properties of *L. paragasseri*, we isolated and here present the draft genomes of three *L. paragasseri* strains.

The three strains were isolated from voided urine samples collected from females with type II diabetes as part of a previous study (IRB# 204197) (4). Strains were isolated using the enhanced quantitative urine culture method (5) and stored at −80°C as part of the Loyola Urinary Education and Research Collaborative (LUEREC) collection. UMB3776 and UMB4347 were isolated from the same participant, with UMB4347 from a collection 3 months later than that producing UMB3776. All three strains were isolated prior to the description of *L. paragasseri* and thus originally identified as *L. gasseri* via MALDI-TOF (6). We obtained the freezer stocks from the collection, and strains were streaked on deMan-Rogosa-Sharpe (MRS) agar plates and incubated under 5% CO_2_ and 35°C for 48 hours. For each strain, a single colony was then put into MRS broth supplemented with 1 mL/L of Tween 80 (Sigma-Aldrich) and incubated as previously described.

DNA extraction was performed using the DNeasy Blood and Tissue Kit (Qiagen) following the manufacturer’s protocol for Gram-positive bacteria. Quantification of DNA was performed utilizing a Qubit fluorometer. The DNA was sent to SeqCenter (Pittsburgh, PA USA) for whole-genome sequencing. There, libraries were prepared using the tagmentation-based and PCR-based Illumina DNA Prep Kit and custom IDT 10 bp unique dual indices with a target insert size of 320 bp. Sequencing was performed on the Illumina NovaSeq 6000 platform (2 × 151 bp paired-end reads). Raw reads were trimmed using BBDuk part of the BBMap suite (v39.01; https://sourceforge.net/projects/bbmap/; parameters: qtrim = rl, ftl = 15, ftr = 135, maq = 20, maxns = 0, and trimq = 20), and filtered reads were assembled using SPAdes v15.5 with the --only-assembler option (7). Genome coverage was calculated using BBWrap (also part of the BBMap suite). Genome assemblies were annotated upon submission to NCBI by the Prokaryotic Genome Annotation Pipeline v6.6 (8).

Average nucleotide identity (ANI) was computed for each assembly using JSpeciesWS and the genome for *L. paragasseri* JCM5343 (9). All three strains had an ANI value greater than or equal to 98.09%, confirming their species assignment. The ANI also was calculated between UMB3776 and UMB4347, and they were found to have an ANI of 99.99%. This suggests that the two strains are closely related.

Next, the three assemblies were screened for biosynthetic gene clusters using antiSmash v7.0.1 with default parameters (10). Gassericin T was identified in all three strains. Furthermore, Gassericin A and Gassericin S were identified in UMB1634. Gassericins have been shown to inhibit the growth of pathogenic bacteria (11). Future testing of these strains is needed to ascertain if they have bacteriostatic or bactericidal properties.

**TABLE 1:** Genome sequencing and sequencing statistics for *Lactobacillus paragasseri* UMB1634, UMB3776, and UMB4347

Strain	No, of raw reads	No. of contigs	Genome length (bp)	N50 (bp)	GC content	Coverage (×)	SRA accession	GenBank assembly accession
UMB1634	2,815,028	42	1,937,262	100,546	34.70%	208.955	SRR26087533	GCA_032377175.1
UMB3776	5,478,692	19	1,919,989	170,775	34.70%	382.611	SRR26087523	GCA_032376475.1
UMB4347	4,910,122	20	1,912,774	231,209	34.70%	340.989	SRR26087525	GCA_032376705.1

## Data Availability

Raw reads and draft assemblies have been deposited in GenBank. Table 1 lists the SRA accession numbers and genome sequencing and assembly accession numbers for all three strains.
